# Smoke-Free Sports in The Netherlands: Why Most Sports Clubs Have Not Adopted an Outdoor Smoke-Free Policy

**DOI:** 10.3390/ijerph18052454

**Published:** 2021-03-02

**Authors:** Heike H. Garritsen, Andrea D. Rozema, Ien A. M. van de Goor, Anton E. Kunst

**Affiliations:** 1 Department of Public and Occupational Health, Amsterdam Public Health Research Institute, Amsterdam UMC, University of Amsterdam, 1105 AZ Amsterdam, The Netherlands; a.e.kunst@amsterdamumc.nl; 2 Tranzo Scientific Center for Care and Wellbeing, Tilburg School of Social and Behavioral Sciences, Tilburg University, 5000 LE Tilburg, The Netherlands; a.d.rozema@tilburguniversity.edu (A.D.R.); l.vandegoor@tilburguniversity.edu (I.A.M.v.d.G.)

**Keywords:** smoke-free, policy, sports, adoption

## Abstract

*Background*: Outdoor smoke-free policies (SFPs) at sports clubs represent an important new area of tobacco control, as many people, including youth, spend a large portion of their free time participating in sports. Nevertheless, the majority of sports clubs worldwide still have not adopted an outdoor SFP. The aim of this study is to explore the perceptions of key stakeholders at different Dutch sports clubs concerning the adoption of an outdoor SFP. *Methods*: Semi-structured interviews were held with 41 key stakeholders at seven Dutch sports clubs (in field hockey, football, tennis, or korfball) without an outdoor SFP. A thematic approach was used to analyze the data. *Results*: The majority of respondents reported considerations that were favorable towards adoption of an outdoor SFP, including expected support from club members, changing social norms with regard to smoking, and few members who smoke. Most of all, respondents valued the protection of children from the harmful effects of smoking. However, they also foresaw a number of problems in case of adoption, including impaired social functioning of the sports club, problems with compliance and enforcement, conflict with smokers’ interest, and low priority in club management. *Conclusions*: Although stakeholders at sports clubs recognize the intrinsic value of an outdoor SFP, they foresee practical problems that are inherent to sports clubs. Adoption could be enhanced by articulating the importance of protecting children from the harmful effects of smoking, referring to ‘success stories’ at sports clubs that are already smoke-free, and actively involving smokers in the adoption process.

## 1. Introduction

Negative health effects associated with exposure to secondhand smoke (SHS) have been well documented [1]. To protect people from the harmful effects of SHS, the WHO Framework Convention on Tobacco Control encouraged countries in 2003 to create smoke-free environments in all indoor workplaces and public places [2]. With scientific evidence demonstrating the hazards of outdoor SHS as well [3], an increasing number of countries have likewise enacted smoke-free policies (SFPs) for outdoor places such as playgrounds, beaches, and outdoor sports facilities [4,5]. The latter has significant reach and potential for prevention of SHS exposure since many people, including youth, spend their free time participating in sports. For example, 12% of European citizens (15 years and over) are member of a sports clubs, with the highest proportions for memberships being observed in the Netherlands (27%) [6].

In recent years, outdoor SFPs at sports clubs have been expanding across Europe as well as internationally, especially in Australia. Nowadays, in the Netherlands, approximately 25% of all (outdoor) sports clubs have voluntarily implemented an outdoor SFP at their venues. However, the majority of sports clubs both in and outside the Netherlands still have not adopted such a policy. A better understanding of policy adoption may help accelerate future SFP adoption at sports clubs.

Little is known about adoption of an outdoor SFP. A few studies have investigated the adoption of a SFP in outdoor settings other than sports clubs, such as recreational facilities, outdoor worksites, and schools [7,8]. Those studies reported factors that facilitate (e.g., sufficient finances and time, education on the harms of smoking) and hinder (e.g., lack of legislation, decision makers being smokers) the adoption of an outdoor SFP. 

Previous results may be difficult to extrapolate to sports clubs since the adoption context may differ. In the Netherlands, sports clubs are voluntary non-profit associations characterized by voluntary membership and services performed by club members. Decisions are made democratically by club members deciding together on a sports club’s policy [9,10]. In addition, sports clubs are important to social integration of communities, and most clubs aim to provide social networks to their members by organizing all kinds of activities and events. In such context, it is important that support for an outdoor SFP is high among club members, and that SFP adoption does not interfere with the club’s dependence on voluntary work and social cohesion.

In order to study this new adoption context, with particular emphasis on the club volunteers, this study aimed to explore the perceptions of key stakeholders at different Dutch sports clubs concerning the adoption of an outdoor SFP. 

## 2. Methods

### 2.1. Participants

Descriptive statistics of the participating sports clubs are presented in Table 1. To represent the variety of Dutch outdoor sports clubs, we included football, tennis, field hockey, and korfball clubs. Sports clubs were also selected to represent different regions and corresponding levels of urbanization. In total, 39 sports clubs were contacted by phone, e-mail, and/or letter, or face-to face and asked whether they would participate. The only inclusion criterion was not having an outdoor SFP. Participating clubs (*n* = 7) did not differ from non-participating clubs (*n* = 32) in type of sports (*p* = 0.66) and urbanity (*p* = 0.93). Main reasons for non-participation were lack of interest (*n* = 16), not having enough respondents (*n* = 7), and being too busy with other things (*n* = 5).

Descriptive statistics of the respondents are presented in Table 2. Semi-structured interviews were held with different stakeholders at each sports club to explore their perceptions with regard to the adoption of an outdoor SFP. Questions were mainly in relationship to respondents’ own sports club, although a number of questions were about adoption of outdoor SFPs at sports clubs in general (e.g., “What hinders implementation of a smoke-free policy?”). The topic guide can be found in Appendix A. To obtain a diverse group of stakeholders, we included 5–6 respondents per sports club who differed in (1) function within the sports club and (2) smoking status (smokers and non-smokers). A total of 41 respondents participated in the study. Their mean age was 43.80 years (SD = 12.57) (range 18–71). Most respondents were committee members (*n* = 15), trainers/coaches (*n* = 12) or board members (*n* = 11). The gender skew toward men might be explained by the fact that in the Netherlands men are more often member of a sports club than women. 

### 2.2. Procedure

The study was conducted in collaboration with Sportief Advies (SA), a Dutch organization that supports non-profit projects with regard to education, sports, and culture. Three employees of SA were responsible for recruiting the sports clubs and conducting the interviews. They were all familiar with the research topic (outdoor SFPs at sports clubs). Because they had little interviewing experience, they were instructed in data collection by the first (HHG) and last (AEK) author. Furthermore, the first author (HHG) provided feedback on their first interviews. Interviews took place at the participating sports clubs, were audio recorded, and lasted on average 17 min (range 6–30). All respondents signed an informed consent form. They did not receive an incentive for their participation. The Medical Ethics Review Committee of the Academic Medical Center confirmed that the Dutch Medical Research Involving Human Subjects Act (WMO) did not apply to this study and that an official approval was not required (letter W20_318 # 20.369).

## 3. Analysis

Interviews were transcribed verbatim by a transcription company and analyzed using MAXQDA [11]. Thematic analysis, a qualitative analytic method for identifying themes within data, was used [12]. Within this thematic analysis, an inductive (or ‘bottom-up’) method was chosen. Coding was conducted by the first author (HHG) and another researcher coded 14 transcripts in parallel. Inconsistencies regarding codes were discussed until consensus was reached. Similar codes were pooled, resulting codes were rearranged, and themes were created based on the final code list. These themes were classified into considerations for and against adoption. In several sessions, the appropriateness of the developed codes and themes were discussed with all authors and amended when necessary. 

## 4. Results

The majority of the respondents reported considerations that were favorable towards the adoption of an outdoor SFP. On the other hand, they also reported considerations against adoption. Below, we first discuss considerations for adoption and then considerations against adoption. 

### 4.1. Considerations for Adoption

#### 4.1.1. Support from Club Members

According to respondents, many people support adoption of an outdoor SFP at their sports club. Some sports clubs conducted a survey to collect people’s opinions about an outdoor SFP. The results of those surveys showed that the majority of club members would like their sports club to become smoke-free. With regard to smokers, respondents mentioned that they expect them to show some resistance in the beginning, but that they will eventually accept the SFP as well. 

Two reasons for support were commonly mentioned. First, according to respondents, it is generally accepted that children should not be exposed to smoking. Children need to be protected from the harmful effects of SHS, and should not be tempted to try a cigarette themselves.

“*In the end, it’s mainly about the young children. They see people smoking and might breathe in smoke themselves. That’s the worst and it should be prevented*”.

(Respondent 11, korfball)

Second, respondents mentioned that smoking is increasingly perceived as being in conflict with the culture of sports clubs. According to them, smoking and sports are opposites, as smoking is unhealthy while participating in sports is healthy. They just do not fit together.

“*Of course, it doesn’t fit. I mean, you’re a sports clubs, and smoking does not fit there*”.

(Respondent 38, korfball)

#### 4.1.2. Change of Social Norm with Regard to Smoking 

Respondents mentioned how, within the society at large, the social norm with regard to smoking has changed. According to them, smoking is a ‘dying habit’, as the number of people who smoke is decreasing. In addition, an increasing number of indoor and outdoor places are smoke-free, such as hospitality venues and hospital grounds. Together, these developments have led to smoking no longer being ‘normal’. Respondents also mentioned that smokers are often already used to the fact that they are not allowed to smoke, making it easier for them to accept the adoption of an outdoor SFP at their sports club.

“*They will think: I can’t smoke at school, I can’t smoke in the car, my roommates don’t allow me to smoke… A smoke-free policy at my sports club might as well be added*”.

(Respondent 31, field hockey)

#### 4.1.3. Few Members Who Smoke

Some sports clubs have only a few members who smoke. According to respondents, this helps to increase support and acceptance with regard to adoption of an outdoor SFP. After all, people who do not smoke themselves will probably not experience any disadvantages of the SFP.

“*I think most people will agree [with the adoption of an outdoor SFP], because the majority of our members do not smoke*”.

(Respondent 11, korfball)

### 4.2. Considerations against Adoption 

#### 4.2.1. Risks to Social Functioning of the Sports Club

According to respondents, as a result of the SFP, people may stay at the sports club for a shorter period of time (e.g., after a match) or even stay away (e.g., parents or other visitors may no longer come to the sports club to watch a game). Some are afraid that this will negatively affect the sports club’s social functioning. First, if people spend less time at the sports club, canteen turnover may decrease. This is worrisome because canteen turnover is an important source of income for sports clubs.

“*I think it’s going to negatively affect our canteen turnover. In that case, where will the money come from? Each year, we have to organize things, pay our trainers, and so on*”.

(Respondent 28, field hockey)

Second, respondents mentioned that sports clubs are afraid of losing smoking volunteers. This is perceived as a major barrier because most sports clubs completely rely on volunteers. That is why, at some clubs, being a smoke-free sports club is considered less important than keeping their volunteers. Nevertheless, most respondents do not expect the SFP to cause people to quit their club membership and move to another, non-smoke-free sports club.

#### 4.2.2. Problems with Compliance and Enforcement

According to respondents, some people expect that smokers will not comply with the SFP and will just continue smoking. In addition, respondents expect difficulties with regard to enforcement. Sports clubs may not have enough volunteers to approach those who continue smoking on club premises. Furthermore, club members may not want to act like ‘police officers’, since they are at the club to enjoy sports together. Consequently, this might result in the SFP not being enforced at all.

“*Who is going to enforce it? When I’m here, I’m here just for fun. I don’t feel like chasing people saying that they can’t smoke here. I’ve got better things to do*”.

(Respondent 26, field hockey)

#### 4.2.3. Low Priority

At some sports clubs, according to respondents, adoption of an outdoor SFP does not have the highest priority. Sports clubs may be too busy with other things, such as attracting new club members or sponsors and organizing all kinds of activities. Furthermore, sports clubs do not always perceive the current situation as intolerable or needing change. A trigger for adopting an outdoor SFP is absent if prominent club members do not perceive the smoking behavior of others as unpleasant.

“*It [smoking] is not a problem at our sports club. I’ve never heard someone complaining about it. If it doesn’t cause any problems, why would you decide to implement a smoke-free policy?*”

(Respondent 9, korfball)

#### 4.2.4. Concern with Smokers’ Interests 

Another consideration against the adoption of an outdoor SFP is that sports clubs want to take into account the interests of all their members, including smokers. Respondents mentioned that they would like everyone to have a good time and that they do not want to exclude people based on their smoking status. In addition, some respondents thought that an outdoor SFP may be considered patronizing.

“*You’re a club, and you want to offend as few people as possible and make sure that everyone is having a good time*”.

(Respondent 26, field hockey)

#### 4.2.5. Obstructing Features of Sports Clubs

Respondents mentioned some obstructing features of sports clubs that can hinder adoption. First, some sports clubs share their property with other sports clubs that are not smoke-free. This may lead to people being confused about the smoking rules, which, in turn, may result in problems with regard to compliance and enforcement. Second, small sports clubs often do not have enough manpower to implement and enforce the policy. Moreover, small clubs may be more vulnerable to possible negative consequences of an outdoor SFP, such as people staying away, volunteers leaving, or declining canteen revenues.

“*Being smoke-free is not a big deal when you’re a field hockey club with over 5000 members. Those clubs will have their income after all. They don’t feel the consequences. But we’re a small club. We need to be very aware of that*”.

(Respondent 28, field hockey)

## 5. Discussion

### 5.1. Key Findings

The aim of the present study was to explore the perceptions of key stakeholders at different sports clubs in the Netherlands concerning the adoption of an outdoor SFP. Most stakeholders in our sample were positive towards adopting an outdoor SFP because of high support among club members and a changing social norm with regard to smoking. Moreover, having few members who smoke facilitates the readiness to adopt. Most of all, stakeholders valued the protection of children from the harmful effects of smoking. On the other hand, stakeholders foresaw various potential problems that may inhibit sports clubs from adopting an outdoor SFP. Fear of risking the club’s social functioning, expected problems with compliance and enforcement, low priority, concern with smokers’ interest, and obstructing features of clubs may prevent sports clubs from undertaking efforts to actually adopt an outdoor SFP. 

### 5.2. Interpretation of Findings

One of the stakeholders’ main reasons to support an outdoor SFP is that children should not be exposed to smoking. This finding is consistent with previous studies on factors facilitating adoption of smoking control policies [13]. A systematic review on public attitudes towards smoke-free outdoor places showed that support was higher for places associated with children, such as school grounds and playgrounds [14]. This implies that support may depend on the degree to which people are aware of the harmful effects of smoking around children and the likelihood of children imitating parents’ and other adults’ smoking behavior. Furthermore, according to the Garbage Can Model [15], decisions in non-profit organizations (such as sports clubs) are more likely to be made when there is a suitable response (solution) to an issue causing concern (problem). This suggests that in order for sports clubs to adopt an outdoor SFP, it is important that such policy is perceived as a solution to the problem of children being exposed to smoking. 

Stakeholders’ fear of losing volunteers may be explained by the fact that the majority of sports clubs rely entirely on the efforts of volunteers [9,10]. Similarly, stakeholders’ concerns about decreasing turnover rates are likely to be related to the fact that the sales of drinks and food is a major source of income for sports clubs. Another concern of stakeholders is that smokers will not to comply with the SFP. However, expected adverse effects of SFPs do not always reflect actual experiences of SFP implementation. For example, a study on outdoor SFPs in parks showed that concerns with compliance were expressed mostly by park directors who had not implemented SFP yet. In contrast, park directors with policy experience portrayed implementation of the outdoor SFP as being relatively easy, and overwhelmingly recommended such policy to other park directors [16]. 

Adoption of an outdoor SFP does not always have the highest priority at sports clubs. It has been suggested that individuals’ shared perceptions of the importance and urgency of an innovation is crucial for adopting it within the organization [17,18]. Since sports clubs have limited time and manpower, choices need to be made regarding which issues to address first. Consequently, issues that are perceived as more urgent may be given priority over adopting an outdoor SFP. 

Stakeholders highly value taking all of their members into account, including smokers. Previous studies found that although many people support SFPs in various outdoor settings [14], some people could strongly oppose outdoor SFPs, arguing these do not respect people’s personal freedom [7,19]. However, instead of being a disadvantage for smokers, outdoor SFPs can support smokers who are trying to quit by limiting their overall cigarette consumption [20].

### 5.3. Potential Limitations

This study is one of the first to disclose the views of stakeholders at sports clubs regarding the adoption of an outdoor SFP. We included a large and diverse group of sports and stakeholders. Nevertheless, some potential limitations should be considered when interpreting the results. First, social desirability bias might have affected the results. Because smoking is increasingly viewed as undesirable behavior, respondents may have felt pressured to say that they support an outdoor smoke-free policy at sports clubs—especially since the interviewers worked for an organization (SA) with a sporty and healthy image. However, despite such bias, we found that most respondents were ready to mention potential problems of an outdoor SFP as well.

A second limitation is that our sample of seven sports clubs was too limited to study differences between clubs. Further study with a larger sample would be needed to examine whether SFP support depends on club characteristics such as geographic location (urban vs. rural), type of sports (field hockey vs. football), or the socioeconomic and ethnic composition of club members.

Third, as ‘lack of interest’ was the primary reason for non-participation, sports clubs that refused to participate in the study might have been those with less positive experiences with an outdoor SFP. Consequently, it could be questioned whether we can fully generalize the findings from our sample to a larger group of sports clubs. 

Finally, some interviews were very short (with one of them lasting only six minutes). This applies mainly to the first interviews and may be explained by the SA employees’ lack of interviewing experience. However, after we provided feedback on their first transcripts and encouraged them to take time to probe for rich data, the duration of the interviews increased.

### 5.4. Implications

The findings of this study have a number of practical implications. First, adoption could be enhanced by articulating that an outdoor SFP at sports club can serve the particular purpose of preventing children from seeing smoking and being exposed to SHS. Second, sports clubs that have successfully implemented an outdoor SFP could be referred to as “success stories”. Although stakeholders at sports clubs recognize the intrinsic value of an outdoor SFP, they foresee practical problems that are inherent to sports clubs. The experiences of sports clubs that are already smoke-free could be documented and their lessons learned could be shared to show that implementation of an outdoor SFP is feasible. Finally, to prevent smokers from feeling patronized or marginalized, sports clubs might consider to involve them at an early stage of the adoption process. Shared-decision making (i.e., collaborating, communicating and involving both smokers and non-smokers) might be essential to increase adoption of an outdoor SFP at sports clubs [8,21]. This is particularly important in settings such as Dutch sports clubs, which are highly dependent on voluntary work and social cohesion.

## 6. Conclusions

This is the first study to explore the adoption of an outdoor SFP at sports clubs. Our findings indicate sports clubs’ willingness to adopt an outdoor SFP. At the same time, sports clubs also express reluctance, as they foresee several problems such adoption may cause. This reluctance could be addressed by articulating the importance to protect children from the harmful effects of smoking, referring to ‘success stories’ at sports clubs that are already smoke-free, and actively involving smokers in the adoption process. Adoption strategies based on these element could be developed and tested in sport clubs worldwide, such that ever more sports clubs are smoke free, and ever more (young) athletes and visitors are protected from SHS. 

## Figures and Tables

**Table 1:** Characteristics of the participating sports clubs.

	No. of Sports Clubs	No. of Respondents
	*n* = 7	
**Sports**		
Football	2	11
Korfball	2	12
Field hockey	2	12
Tennis	1	6
**Size**		
<250 members	3	18
250–500 members	0	0
500–1000 members	3	18
1000–1500 members	1	5
**Degree of urbanization**		
Very high	1	5
High	0	0
Moderately	1	6
Low	4	24
Rural	1	6

**Table 2:** Characteristics of the respondents.

	No. of Respondents	%
	*n* = 41	
**Gender**		
Men	25	61.0
Women	16	39.0
**Function** ^a^		
Committee member ^b^	15	36.6
Trainer/coach	12	29.3
Board member	11	26.8
Other ^c^	8	19.5
Arbitrator	3	7.3
Parent	2	4.9
**Smoking status** ^d^		
Smoker	6	14.6
Non-smoker	35	85.4

^a^ Total numbers in rows may not add up to 41 as some respondents had multiple functions within the sports club. ^b^ Includes committees such as the bar committee, technical committee, youth committee, tournament committee and party committee. ^c^ Includes volunteers with other tasks, such as a website builder and a secretary. ^d^ Smoker refers to daily smokers; non-smoker refers to non-daily smokers and non-smokers.

## Appendix A

1. How would you describe the sports club?

2. What topics are currently being discussed at the sports club?

2.b. And what about smoking?

3. To what extent do people smoke at your sports club?

3.a. When do people smoke?

3.b. Where do people smoke?

3.c. Who is smoking?

4. What rules does this sports club have for smoking on the sports grounds?

4.a. Where do these rules apply?

4.b. When do these rules apply?

4.c. To whom do these rules apply?

4.d. What are the rules at parties, tournaments, or other events?

4.e. What are the rules for the e-cigarette?

5. What do you think about the smoking rules at your sports club?

6. What do other members think about these rules?

7. Does the sports club currently have plans to change the smoking rules?

YES:  - Why?

- What do these plans look like?

NO:  - Do you expect the sports club to change the smoking rules within the next two years?

- Can you explain this?

8. What hinders implementation of a smoke-free policy?

8.a. Are there other things that hinder implementation?

8.b. Are there other things?

NB: Keep on asking questions until all factors have been identified

9. What facilitates implementation of a smoke-free policy?

9.a. Are there other things that facilitate implementation?

9.b. Are there other things?

NB: Keep on asking questions until all factors have been identified

10. Which parties outside the sports club have an influence on the smoking policy of a sports club?

10.a. Can you explain this?

10.b. Are there more parties important, besides party X?

10.c. Are there more parties important?

NB: Keep on asking questions until all factors have been identified

11. Imagine that your sports club implements a smoke-free policy. How will this turn out?

11.a. How do you expect club members to react?

## References

[1] World Health Organization (2008). Second-Hand Smoke: Assessing the Burden of Disease at National and Local Levels.

[2] World Health Organization (2003). WHO Framework Convention on Tobacco Control.

[3] Eriksen M.P., Cerak R.L. (2008). The diffusion and impact of clean indoor air laws. Annu. Rev. Public Health.

[4] Joossens L., Feliu A., Fernandez E. The Tobacco Control Scale 2019 in Europe. http://www.tobaccocontrolscale.org/TCS2019.pdf.

[5] World Health Organization (2018). Global Progress Report on Implementation of the WHO Framework Convention on Tobacco Control. https://www.who.int/fctc/reporting/WHO-FCTC-2018_global_progress_report.pdf?ua=1.

[6] European Union Special Eurobarometer 472. Sport and Physical Activity. https://ec.europa.eu/sport/news/2018/new-eurobarometer-sport-and-physical-activity_en.

[7] Satterlund T.D., Cassady D., Treinber J., Lemp C. (2011). Barriers to adopting and implementing local-level tobacco control policies. J. Community Health.

[8] Rozema A.D., Mathijssen J.J.P., Jansen M.W.J., van Oers J.A.M. (2016). Schools as smoke-free zones? Barriers and facilitators to the adoption of outdoor school ground smoking bans at secondary schools. Tob. Induc. Dis..

[9] Thiel A., Mayer J. (2009). Characteristics of voluntary sports clubs management: A sociological perspective. Eur. Sport Manag. Q.

[10] Lucassen J.M.H., van der Roest J., Brandsema A., Davids A., Elling A., ter Haar M. Sportverenigingen in Nederland: Veerkrachtige Verbanden Voor Sport, Brancherapport Sport. https://www.kenniscentrumsportenbewegen.nl/kennisbank/publicaties/?sportverenigingen-in-nederland&kb_id=24389.

[11] VERBI Software (2020). MAXQDA Analytics Pro.

[12] Braun V., Clarke V. (2006). Using thematic analysis in psychology. Qual. Res. Psychol..

[13] Kuijpers T.G., Willemsen M.C., Kunst A.E. (2018). Public support for tobacco control policies: The role of the protection of children against tobacco. Health Policy.

[14] Thomson G., Wilson N., Edwards R. (2009). At the frontier of tobacco control: A brief review of public attitudes toward smoke-free outdoor places. Nic. Tob. Res..

[15] Cohen M.D., March J.G., Olsen J.P. (1972). A garbage can model of organizational choice. Adm. Sci. Q..

[16] Klein E.G., Forster J.L., McFadden B., Outley C.W. (2007). Minnesota tobacco-free park policies: Attitudes of the general public and park officials. Nic. Tob. Res..

[17] Helfrich C.D., Weiner B.J., McKinney M.M., Minasian L. (2007). Determinants of implementation effectiveness: Adapting a framework for complex innovations. Med. Care Res. Rev..

[18] Klein K.J., Conn A.B., Sorra J.S. (2001). Implementing computerized technology: An organizational analysis. J. Appl. Psychol..

[19] Chapman S. (2000). Banning smoking outdoors is seldom ethically justifiable. Tob. Control..

[20] Williams S.C., Hafner J.M., Morton D.J. (2009). The adoption of smoke-free hospital campuses in the United States. Tob. Control..

[21] Durlak J.A., DuPre E.P. (2008). Implementation matters: A review of research on the influence of implementation on program outcomes and the factors affecting implementation. Am. J. Community Psychol..

